# A simulation study of three methods for detecting disease clusters

**DOI:** 10.1186/1476-072X-5-15

**Published:** 2006-04-12

**Authors:** Geir Aamodt, Sven O Samuelsen, Anders Skrondal

**Affiliations:** 1 Akershus University Hospital, University of Oslo, Norway; 2 Department of Mathematics, University of Oslo, Norway; 3 Division of Epidemiology, Norwegian Institute of Public Health, Norway; 4 Department of Statistics, London School of Economics, UK

## Abstract

**Background:**

Cluster detection is an important part of spatial epidemiology because it can help identifying environmental factors associated with disease and thus guide investigation of the aetiology of diseases. In this article we study three methods suitable for detecting local spatial clusters: (1) a spatial scan statistic (SaTScan), (2) generalized additive models (GAM) and (3) Bayesian disease mapping (BYM). We conducted a simulation study to compare the methods. Seven geographic clusters with different shapes were initially chosen as high-risk areas. Different scenarios for the magnitude of the relative risk of these areas as compared to the normal risk areas were considered. For each scenario the performance of the methods were assessed in terms of the sensitivity, specificity, and percentage correctly classified for each cluster.

**Results:**

The performance depends on the relative risk, but all methods are in general suitable for identifying clusters with a relative risk larger than 1.5. However, it is difficult to detect clusters with lower relative risks. The GAM approach had the highest sensitivity, but relatively low specificity leading to an overestimation of the cluster area. Both the BYM and the SaTScan methods work well. Clusters with irregular shapes are more difficult to detect than more circular clusters.

**Conclusion:**

Based on our simulations we conclude that the methods differ in their ability to detect spatial clusters. Different aspects should be considered for appropriate choice of method such as size and shape of the assumed spatial clusters and the relative importance of sensitivity and specificity. In general, the BYM method seems preferable for local cluster detection with relatively high relative risks whereas the SaTScan method appears preferable for lower relative risks. The GAM method needs to be tuned (using cross-validation) to get satisfactory results.

## Background

A spatial cluster is defined as a limited area within the general study area with a significant increase in the incidence of a disease (a hot-spot cluster) [1], p. 104. The identification of a cluster of disease can help epidemiologists determining putative environmental risk factors and lead to improved understanding of aetiology. Furthermore, identification of clusters facilitates more detailed investigations using case-control studies to estimate the association between exposures and disease or targeted interventions. For example, in a study of sexually transmitted infections [2], eight clusters with high risk of gonorrhoea were identified, which were subsequently subject to intervention.

Different methods have been proposed to locate and identify the clusters dependent on whether the locations of the clusters are suspected or known (focused) or unknown (non-focused). Models for focused clusters are designed for detecting preconceived patterns linked to objects such as power lines or putative sources such as landfill sites [3,4]. Models for non-focused clusters, on the other hand, are designed to estimate the relative risk for each area within the study area. Typically, these models accommodate extra-Poisson variability in different ways [5-7].

Methods also exist to identify global clusters where the presence – but not location – of the clusters is of interest. Examples of such methods are the maximizing excess events test [8], the Bonetti-Pagano M statistic[9], and the spatial scan statistic [10]. The purpose of these methods is to test the null hypothesis of no spatial clustering. The spatial scan statistic is applicable for both global tests and non-focused cluster detection.

In this study we evaluate three different methods with potential for detecting and identifying local cluster patterns for count data, i.e. the number of cases for each area or municipality. The main outcome measure is the estimated relative risk for each municipality or a neighbourhood of municipalities in the study area. Additional explanatory variables are not included in the comparisons. Methods also exist for case event data where the location given as coordinates for each individual is known [1].

Simulation has been used to evaluate and compare statistical power for different global cluster detection methods [11,12] as well as for Bayesian methods [13,14], but little is known concerning their ability to detect different patterns of clusters. The patterns or shapes of disease clusters may vary due to their origin, and it is likely that some methods are more suited to detect specific cluster morphologies than others.

The purpose of this article is to evaluate the performance of three methods for local cluster detection for different types of spatial clusters. The work was motivated by the desire to study the relationship between different disease characteristics, such as the shape of clusters and the risk ratio between high-risk and normal risk areas, and the methods used to detect and depict spatial clusters. The methods differ in the way they detect clusters: SaTScan is based on a likelihood-ratio test to identify areas with increased incidences, whereas BYM and GAM are based on estimation of relative risks. However, all three methods are applicable to detect hot-spot clusters. The ability to detect the different types of clusters is assessed by the sensitivity, specificity and percentage of correct classification.

## Methods

### Spatial scan statistic (SaTScan)

The SaTScan method [10] is based on a spatial scan statistic and is used in an increasing number of applications both within the field of epidemiology [15] and in other research fields. The method works as follows: circles of different sizes (from zero up to 50 % of the population size) are placed at every municipality in Norway. For each circle a likelihood ratio statistic is computed based on the number of observed and expected cases within and outside the circle and compared with the likelihood *L*_*0 *_under the null hypothesis. The likelihood function under the alternative hypothesis assuming Poisson distributed cases is proportional to:



The symbols *y *and *E(y) *represent the observed and expected number of cases in a circle and *(N-y) *and *(N-E(y)) *the observed and expected number of cases outside the circle. *N *is the total number of cases. The indicator function *I() *is equal to 1 if the observed number of cases within the circle is larger than the expected number of cases given the null hypothesis and 0 otherwise. The circles with the highest likelihood ratio values are identified as potential clusters. An associated p-value, based on Monte Carlo simulations, is computed and used to evaluate whether the cases are randomly distributed in space or not. For each simulation the likelihood ratio statistic is computed and the actual value is compared with the set of simulated values to find the significance probability.

The method produces a set of clusters, the relative risk of disease for the different clusters, and a corresponding p-value for each cluster based on the Monte-Carlo simulations. Municipalities are identified as clusters if they are associated with a cluster with a p-value less than 0.05. In addition to the number of cases in each municipality, the population and coordinates for the geographic centre of the municipalities must be given. To accommodate different at-risk groups, such as gender and age intervals, the population can be split into different strata. The model is designed for spatio-temporal problems, but explanatory variables, assumed to be associated with the clustering, cannot be included. We used the free software SaTScan ™ to find the areas with increased relative risks. The SaTScan program also accommodates binomial probability models for the observed cases.

In the present study, we included both primary and secondary clusters, as long as their corresponding p-values were less than 0.05. The p-values are based on 999 Monte Carlo simulations for each dataset, as suggested by Kulldorff [10]. The maximum cluster size was set to 50% of the population size.

### Generalized additive models (GAM)

Generalised additive models were first introduced by Hastie and Tibshirani [16] and are used in a variety of settings including cluster detection of diseases [17,18]. A smooth function *f(s*_*i*_*) *with *s*_*i *_= *(x*_*i1*_*, x*_*i2*_*) *as coordinates is fitted, corresponding to a latent spatial process of the incidences. If *y*_*i *_and *n*_*i *_are the number of cases and persons at-risk at site *i*, respectively, we get:

log(*E*[*y*_*i*_]) = log(*n*_*i*_) + *f*(*s*_*i*_).

We define a cluster as an area in which the lower limit of the 95% confidence interval for the estimated relative risk is above 1.0. In epidemiological settings the observed values are assumed to follow a Poisson distribution. In our example we used a local regression smoother (loess) [19], which is an integrated part of the Splus software. The degree of smoothness is governed by the span parameter and we used the default value of 0.75 in our study.

### Disease mapping model (BYM)

The BYM model, named after Besag, York and Mollie [5], was originally developed for pattern recognition. The model has also been used in spatial epidemiology [20] for disease mapping and in ecological studies [21]. The model consists of two parts. In the first part the cases are assumed to follow a Poisson distribution with an area specific parameter *φ*_*i*_.

*y*_*i *_~ *Poisson*(*φ*_*i*_).

The second part is a log-linear model with a grand mean *α*, an offset *log(n*_*i*_*)*, an uncorrelated component *v*_*i*_, and a correlated component *u*_*i*_,

log(φ_*i*_) = log(*n*_*i*_) + α + *v*_*i *_+ *u*_*i*_.

The two last terms accommodates the extra-Poisson variability. The uncorrelated component is assumed to follow a normal distribution with zero mean and a common variance *σ*_*v*_^*2*^. The correlated component is assumed to follow a conditional autoregressive distribution dependent on their neighbouring values. Following Besag et al[5], the variance σ_u_^2 ^for this term is computed as a weighting of the standard deviation for the adjacent areas. The parameters can be estimated within the Bayesian framework using non-informative priors for the parameters [5]. This produces posterior distributions for the parameters in the model.

A cluster is defined as an area where the estimated relative risk is "significantly" (in terms of their credibility sets) larger than 1. Explanatory variables are easily included, but the facilities for spatio-temporal model extensions to detect local clusters in space and time are not comparable to the SaTScan model. We used the free WinBugs ™ software to compute the relative risk values and their credibility sets. A burn-in period of 3000 iterations was used and an additional 3000 simulations were conducted to estimate the posterior distributions.

### Other differences between the methods

The three methods also differ when data is given as case events with coordinates assumed to reflect the location of the exposure for each individual. SaTScan can easily include such data in contrast to the GAM and BYM methods.

### Design of the simulation study

We used the 2002 official Norwegian population statistics as the basis for our simulations. The total population size in Norway was 4577457 inhabitants. The country is divided into 434 municipalities, which constitute the lowest administrative unit in Norway. The municipalities vary considerably as regards economic and social characteristics. The 25 percentile, median, and 75 percentile of the population sizes are 2273, 4400, and 9225 inhabitants, respectively. Geographic Information System (GIS) is a standard tool in regional planning, as well as in infectious disease epidemiology in Norway.

**Figure 1:**
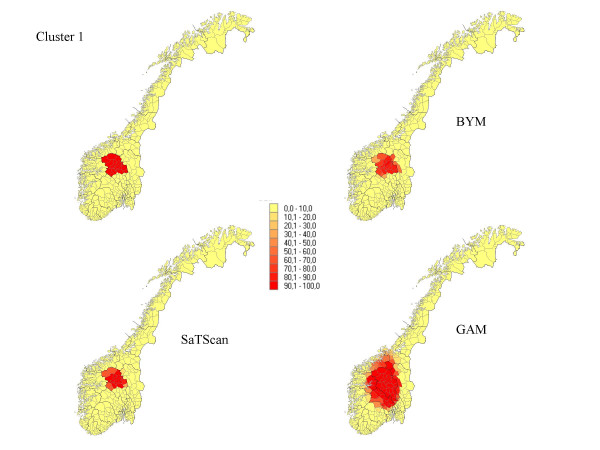
The percentage of each municipality classified as a high-risk municipality for a relative risk of 2.5 for cluster 1

Seven different clusters where produced based on the 434 municipalities: (1) 14 municipalities in a circular pattern (1.1% of the total population) from the middle part of Norway, (2) 6 municipalities along a Norwegian river (1.6% of the population), (3) 15 municipalities (5.2% of the population) along the Norwegian southern coast, (4) 4 separated clusters from 70 municipalities (13.0 % of the population), (5) division into an area of high risk in the south (345 municipalities, 89.9 % of the population) and low risk in the north (89 municipalities, 10.1 % of the population), (6) a cluster in the southern part of the country (125 municipalities, 32 % of the population), and (7) a case of no clusters. The different cluster patterns are shown in the upper left picture of Figure 1 to Figure 7.

**Figure 2:**
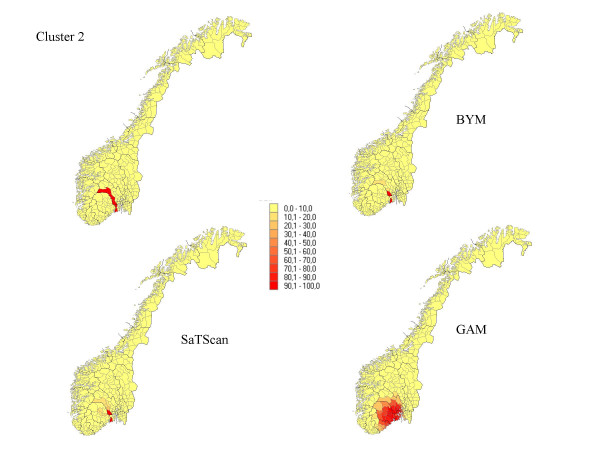
The percentage of each municipality classified as a high-risk municipality for a relative risk of 2.5 for cluster 2.

**Figure 3:**
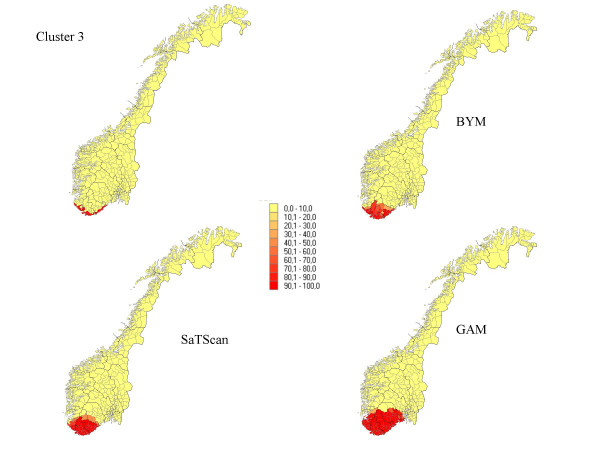
The percentage of each municipality classified as a high-risk municipality for a relative risk of 2.5 for cluster 3.

**Figure 4:**
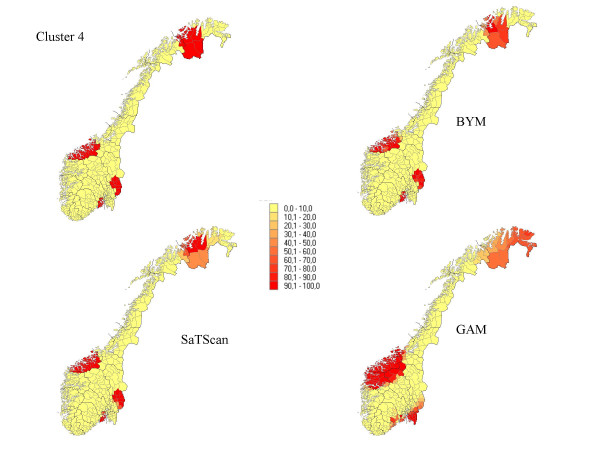
The percentage of each municipality classified as a high-risk municipality for a relative risk of 2.5 for cluster 4.

**Figure 5:**
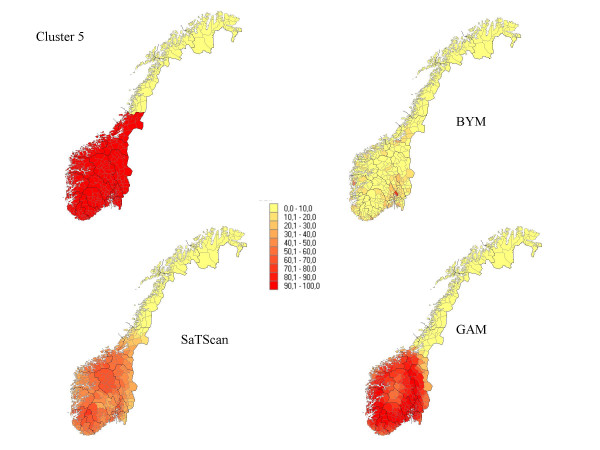
The percentage of each municipality classified as a high-risk municipality for a relative risk of 2.5 for cluster 5.

**Figure 6:**
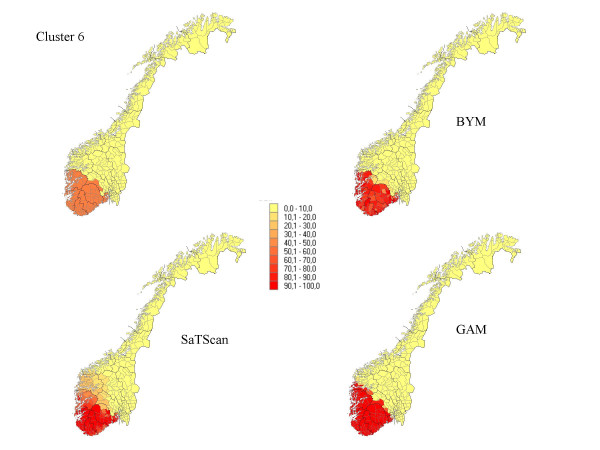
The percentage of each municipality classified as a high-risk municipality for a relative risk of 2.5 for cluster 6.

**Figure 7:**
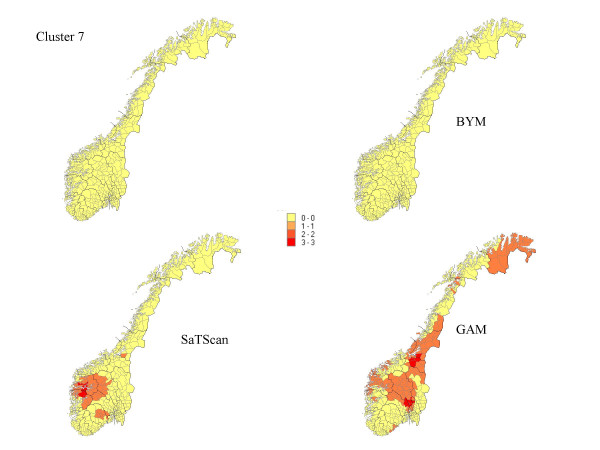
The percentage of each municipality classified as a high-risk municipality for the scenario of no high-risk municipalities.

500 simulated datasets were produced for each combination of cluster pattern and magnitude of relative risk. For each combination the sensitivity is estimated as the average percentage of high-risk municipalities that are correctly classified as high-risk municipalities. The specificity is estimated as the average percentage of normal risk municipalities that are correctly classified as normal risk municipalities. We also computed the average percentage of municipalities that are correctly classified as both normal risk and high-risk municipalities.

The data were simulated from Poisson distributions. The expected number of cases was 0.2% of the background population, which corresponds to an incidence rate of 200 cases per 100000 inhabitants. We repeated the simulation procedure with five different values for the relative risk of disease between the high-risk municipalities and the normal risk municipalities. The magnitudes of the relative risks were 1.2, 1.5, 2.5, 4 and 10. Wilcoxon's signed rank test was used to compare the performance of pairs of methods.

## Results

The results from our simulations are summarised in Table 1, which contains the sensitivity, specificity, and the percentage correctly classified municipalities for the different cluster types and magnitudes of relative risk. In Figures 1 to Figure 7, the percentage of each municipality classified as a high-risk area is depicted for a relative risk of 2.5. In general, the GAM method shows satisfactory sensitivity but relatively low specificity. For the three small clusters the specificities are around 80–90%, but since the number of municipalities in the clusters is small (6–15), the cluster sizes are overestimated by a factor of two (see lower right graphs in Figure 1 to Figure 7).

**Table 1:** Sensitivity, specificity and percentage correct classification (CC) for SaTScan, BYM and GAM based on 500 replications

		SaTScan			BYM			GAM		
				
RR	Cluster	Sensitivity	Specificity	CC	Sensitivity	Specificity	CC	Sensitivity	Specificity	CC
10	1	89.3 %	99.9 %	99.6 %	99.3 %	99.3 %	99.3 %	100.0 %	75.5 %	76.3 %
	2	65.0 %	97.8 %	97.4 %	95.0 %	99.9 %	99.9 %	100.0 %	79.3 %	79.6 %
	3	99.3 %	94.6 %	94.8 %	100.0 %	99.9 %	99.9 %	100.0 %	86.5 %	86.9 %
	4	92.0 %	99.0 %	97.9 %	97.1 %	100.0 %	99.5 %	83.6 %	75.2 %	76.5 %
	5	63.6 %	100.0 %	71.1 %	37.0 %	100.0 %	49.9 %	90.8 %	100.0 %	92.7 %
	6	81.3 %	96.3 %	92.0 %	90.6 %	100.0 %	97.3 %	92.2 %	97.6 %	96.1 %

4	1	87.1 %	99.9 %	99.5 %	98.6 %	99.0 %	99.0 %	100.0 %	85.6 %	86.1 %
	2	43.3 %	99.6 %	98.8 %	73.3 %	99.9 %	99.5 %	71.7 %	84.9 %	84.7 %
	3	98.0 %	94.9 %	95.0 %	100.0 %	98.4 %	98.5 %	100.0 %	86.5 %	87.0 %
	4	91.1 %	99.2 %	97.9 %	93.0 %	100.0 %	98.8 %	82.6 %	77.6 %	78.4 %
	5	59.2 %	100.0 %	67.6 %	20.1 %	100.0 %	36.5 %	87.6 %	100.0 %	90.1 %
	6	80.1 %	96.5 %	91.8 %	84.7 %	100.0 %	95.6 %	93.9 %	97.1 %	96.2 %

2.5	1	83.6 %	99.8 %	99.3 %	77.9 %	99.5 %	98.8 %	99.3 %	89.0 %	89.3 %
	2	43.3 %	99.7 %	98.9 %	41.7 %	100.0 %	99.1 %	71.7 %	89.6 %	89.4 %
	3	96.0 %	95.3 %	95.3 %	98.7 %	97.1 %	97.2 %	100.0 %	86.9 %	87.4 %
	4	89.0 %	99.2 %	97.5 %	85.3 %	99.9 %	97.5 %	69.0 %	82.2 %	80.1 %
	5	47.2 %	100.0 %	58.0 %	9.4 %	100.0 %	28.0 %	73.3 %	100.0 %	78.8 %
	6	78.2 %	96.8 %	91.5 %	77.9 %	100.0 %	93.6 %	94.7 %	97.1 %	96.4 %

1.5	1	50.7 %	99.2 %	97.7 %	0.0 %	100.0 %	96.8 %	21.4 %	97.7 %	95.3 %
	2	20.0 %	99.2 %	98.1 %	8.3 %	100.0 %	98.7 %	6.7 %	98.7 %	97.4 %
	3	86.0 %	95.7 %	95.3 %	71.3 %	97.9 %	97.0 %	98.0 %	91.4 %	91.6 %
	4	71.0 %	98.8 %	94.3 %	45.3 %	99.9 %	91.1 %	56.3 %	92.4 %	86.5 %
	5	16.0 %	100.0 %	33.2 %	1.0 %	100.0 %	21.3 %	27.2 %	100.0 %	42.1 %
	6	74.6 %	97.3 %	90.8 %	51.3 %	100.0 %	86.0 %	90.4 %	98.3 %	96.1 %

1.2	1	4.3 %	99.5 %	96.4 %	0.0 %	100.0 %	96.8 %	2.9 %	99.1 %	96.0 %
	2	3.3 %	99.6 %	98.3 %	0.0 %	100.0 %	98.6 %	1.7 %	99.3 %	98.0 %
	3	34.7 %	97.8 %	95.6 %	5.3 %	100.0 %	96.7 %	70.0 %	96.1 %	95.2 %
	4	20.1 %	98.8 %	86.2 %	1.6 %	100.0 %	84.1 %	29.3 %	98.3 %	87.2 %
	5	2.3 %	100.0 %	22.3 %	0.1 %	100.0 %	20.6 %	5.6 %	100.0 %	25.0 %
	6	56.4 %	99.4 %	87.0 %	9.3 %	100.0 %	73.9 %	71.7 %	99.6 %	91.6 %

1	7	100.0 %	99.9 %	99.9 %	100.0 %	100.0 %	100 %	100.0 %	99.5 %	99.5 %

The BYM method has higher sensitivity than the SaTScan method for relative risks larger than 2.5 for all cluster types except cluster type 5 (p < 0.05). SaTScan has higher sensitivity than BYM for relative risks less than 2.5 (p < 0.05). For a relative risk of 2.5 SaTScan has higher sensitivity than BYM for cluster types 4 and 5 (p < 0.05), BYM has higher sensitivity than SaTScan for cluster type 3 (p < 0.05), but no significant differences are observed for cluster types 1, 2, and 6. The mean values of sensitivity change dramatically for relative risks of 1.5 (44%) and 1.2 (18%) as compared to the three higher magnitudes of relative risk (88%, 81% and 74%).

The second cluster, which has a narrow and long shape, is the most difficult cluster to detect for all methods and almost all values of relative risk. The third cluster is, somewhat surprisingly, the one with the highest sensitivity yet high specificity. It is also surprising that the second and third clusters and the first and fourth clusters do not show the same properties in spite of having approximately similar shape. For relative risks larger than 1.5, it is evident that large clusters are more difficult to detect than small clusters, having high specificity but dismal sensitivity. The sixth cluster includes 89.9% of the population size and exceeds the maximal cluster size for the SaTScan method. Nevertheless, the SaTScan method showed better performance than the BYM model, but worse than the GAM method for this cluster.

We also included a no-cluster scenario (Figure 7). This scenario produces the false alarm rates for the methods. There were no false alarms for the BYM method, but some scattered false alarms for the SaTScan and the GAM methods (Table 1, bottom line). As shown in Table 1, the specificity is higher for the BYM method than for the GAM and SaTScan methods for all cluster types and magnitudes of relative risks except for the first cluster type with relative risk equal to 2.5, 4, and 10.

## Discussion

The three methods considered in this article, SaTScan, BYM and GAM, are different in their model specifications and it seems futile to attempt explaining their different performance analytically. Instead, we identify some differences that might explain their performance.

The satisfactory performance of the SaTScan method concurs with Song et al. who reported high power in their study [12]. The relative performance of generalized additive models (GAM) and the Bayesian method is in accordance with Lawson et al. [14], although the performance of GAM can be significantly improved with a proper tuning of the smoothing parameter (results not shown). Kelsall and Diggle used cross-validation to perform the tuning [17]. However, little is previously known about the properties investigated in the present study such as ability to correctly identify clusters of high-risk municipalities.

The shape of the preconceived pattern of a cluster is the factor producing the most pronounced differences in performance for the three methods. The SaTScan method requires a circular pattern of the cluster with the likelihood ratio statistic computed based on the number of expected and observed cases both within and outside this circular cluster. The BYM and the GAM methods basically smooth the data. The correlated component in BYM is dependent on adjacent areas but in a more flexible way than for the SaTScan method. It is therefore likely that the methods should have similar performance for circular clusters such as our first pattern. Conversely, the BYM method would be expected to have higher sensitivity for banana shaped clusters such as patterns 2 and 3. None of the methods are suitable for detecting borders between high-risk and normal risk areas. For a given group of high-risk municipalities, the three methods will force the surrounding municipalities into the cluster, either due to a fixed circular cluster pattern or due to smoothing. Alternatively, we will lose some of the high-risk municipalities in the cluster. This topic is not studied in detail.

The SaTScan method accommodates the problem of multiple testing in contrast to the other methods. This problem is not relevant in our studies because we focus on detecting high-risk municipalities and not on determining the number of clusters needed to detect high-risk areas.

The methods differ in their use of the geographic locations of the municipalities and their neighbouring municipalities. The circular scan performed in the SaTScan method is independent on whether the municipalities within the circles are neighbours or not. In contrast, for the BYM method the smoothing in the correlated term is dependent on the neighbouring municipalities only, regardless of the distance between them and their population. It is therefore likely that SaTScan might detect larger clusters then the BYM method. Indeed, this was what we observed in our study. An interesting method called flexible shaped spatial scan statistic has recently been proposed [22], which allows clusters to be of a more general shape.

There are also some practical differences between the methods. The SaTScan and GAM methods are relatively simple to implement. For each municipality the coordinates for the centre are used in addition to the population and number of cases in each municipality. To specify the BYM model correctly it is necessary to find the adjacent municipalities for all municipalities in the study. This operation is both time-consuming and expensive but is an integrated part of the Bugs software once the polygons of each municipality are given. The model fitting takes slightly more time for BYM than for the SaTScan method.

The choice of the appropriate method to detect local clusters in a new study should be based on different considerations. These are: (1) the assumed size and shape of the expected disease clusters and type of mode of disease transmission, (2) the assumed ratio between incidence rate in high-risk compared to normal risk areas, and (3) the trade-off between sensitivity or specificity. First, diseases related to water-born pollution and diseases related to pollution from traffic are often irregular in shape and are likely to be best studied with the BYM method. Circular shaped clusters and clusters caused by regional aerosol contamination are better studied with the SaTScan or GAM methods. An example of the latter is the Chernobyl accident and its pattern of fallout in a country like Norway [23]. Second, if high specificity (a low false alarm rate) is valued more than high sensitivity, the BYM method is better than the SaTScan and GAM methods, or visa versa if high sensitivity is more important than a low false alarm rate. Third, if the relative risk between high-risk and normal risk areas is small, the SaTScan method is better than BYM and GAM methods.

Our study is for simplicity confined to spatial clusters. Perhaps of more interest are clusters in both space and time where we want to identify specific areas and points in time. The SaTScan method is targeted for such problems in contrast to the BYM method. Space and time versions exist for this model, but they are not as geared to identifying space-time clusters as SaTScan. We have included only spatially correlated extra-Poisson variability or overdispersion in our simulations. It is possible that the performance of the methods would deteriorate if we were to include uncorrelated extra-Poisson variability as well. The GAM and BYM methods handle both types of overdispersion in contrast to the SaTScan method. For GAM as implemented in Splus the 'quasi' link function together with a proper variance function accommodates overdispersion.

The present study is based on Norwegian conditions, with its shape, size and population. The shape, size and population of the 434 municipalities also vary and it is likely that this influenced the results in our study and introduced some degrees of bias.

## Conclusion

Based on our simulations we conclude that the BYM method seems preferable for local cluster detection with relatively high relative risks whereas the SaTScan method appears preferable for lower relative risks. The assumed shape and size of the clusters is also of importance. The GAM method needs to be tuned to get satisfactory results. None of the methods are able to detect clusters with low relative risks (magnitudes less than 1.5).

## Competing interests

The author(s) declare that they have no competing interests.

## Authors' contributions

GA, SOS and AS jointly conceived and designed the study. GA programmed the Splus code and administered the simulations. All authors interpreted the results and wrote the paper.
